# Effects of High-Dose Prednisone on the Gastrointestinal Microbiota of Healthy Dogs

**DOI:** 10.3390/vetsci12030216

**Published:** 2025-03-02

**Authors:** Sarah Garrity, Jacqueline C. Whittemore, Dipak Kumar Sahoo, Shannon Morgan, Emily Lindgreen, Sarah VanDeWalle, Jan S. Suchodolski, Albert E. Jergens

**Affiliations:** 1 The Department of Small Animal Clinical Sciences, University of Tennessee College of Veterinary Medicine, Knoxville, TN 37996, USA; sgarrity@utk.edu (S.G.); jwhittemore@utk.edu (J.C.W.); 2 Department of Veterinary Clinical Sciences, College of Veterinary Medicine, Iowa State University, Ames, IA 50011, USA; dsahoo@iastate.edu (D.K.S.); smmorgan@iastate.edu (S.M.); lindgree@iastate.edu (E.L.); sev@iastate.edu (S.V.); 3 Gastrointestinal Laboratory, School of Veterinary Medicine and Biomedical Sciences, Texas A&M University, College Station, TX 77843, USA; jsuchodolski@cvm.tamu.edu

**Keywords:** dog, prednisone, microbiota, fluorescence in situ hybridization, gastrointestinal, glucocorticoid, dysbiosis index

## Abstract

The effects of high-dose glucocorticoids on the gastrointestinal microbiota of healthy dogs are unknown. This study’s aim was to investigate the effects of immunosuppressive doses of prednisone on the fecal microbiota and the gastric and duodenal mucosal microbiota in healthy dogs. Twelve healthy adult dogs were enrolled into a randomized, double-blinded, placebo-controlled trial. Dogs were evaluated on days 0, 14, and 28 following treatments with either prednisone (2 mg/kg/d) or placebo. Outcome measures included (1) composition and abundance of the fecal microbiota (via high-throughput sequencing of the 16S rRNA gene and qPCR-based dysbiosis index [DI]) and (2) spatial distribution of the gastric and duodenal mucosal microbiota using fluorescence in situ hybridization (FISH). No significant difference in alpha and beta diversity or amplicon sequence variants of the fecal microbiota was observed between treatment groups. *Blautia* spp. concentrations via qPCR were significantly decreased between prednisone group timepoints 2 and 3. Compared to placebo group dogs, prednisone group dogs showed significantly increased gastric mucosal helicobacters and increased mucosal-associated total bacteria and Bacteroides in duodenal biopsies over the treatment period. The results indicate that immunosuppressive dosages of prednisone alter the mucosal microbiota of healthy dogs in a time-dependent manner, which may disrupt mucosal homeostasis. This report is significant, since it addresses a knowledge gap in our understanding of the effects of glucocorticoids on the gastrointestinal mucosal microbiota of healthy dogs.

## Simple Summary

It is unknown what happens to healthy dogs’ gastrointestinal microbes when administering high-dose glucocorticoids. This randomized, double-blinded, placebo-controlled trial examined the mucosal microbiota present within gastric and duodenal endoscopic biopsies of twelve healthy dogs following 28 days of immunosuppressive therapy using prednisone. The dogs were evaluated on days 0 (pre-treatment) and 14 and 28 days after receiving prednisone (2 mg/kg/day PO) or a placebo (PO). Fluorescence in situ hybridization (FISH) and high-throughput sequencing (Illumina, San Diego, CA, CA) were performed to evaluate select bacterial groups in GI tissues and feces at each treatment timepoint (days 0, 14, and 28). The results showed that there was little change in the fecal microbiota, except for decreased *Blautia* spp. at timepoint 3. However, the prednisone group dogs showed increased gastric mucosal helicobacters and increased mucosal total bacteria and Bacteroides in duodenal biopsies over the 28-day treatment period, as compared to placebo group dogs. These findings indicate that immunosuppressive dosages of prednisone alter the mucosal microbiota of healthy dogs in a time-dependent manner. This report is significant, since it addresses a knowledge gap in our understanding of how glucocorticoids disturb the gastrointestinal mucosal microbiota of healthy dogs.

## 1. Introduction

Glucocorticoids (GCs) are used clinically for their anti-inflammatory and immunosuppressive actions but are associated with multiple side effects [1,2,3]. Common side effects include polydipsia, polyuria, vomiting, and diarrhea [4]. High-dose glucocorticoid therapy may cause gastric erosion and ulceration in healthy dogs and people [5,6,7,8]. The mechanisms of action for GC-induced injury include decreased gastric emptying via prostaglandin E2 deficiency, gastric hyperacidity, and oxidative injury to the gastric mucosa [5,7,9]. Moreover, GC-induced gastric mucosal injury may disrupt normal gastric emptying and predispose one to alterations in the gastrointestinal (GI) microbiota that are different than eubiosis observed in GI health [7].

Despite their frequent use by clinicians to treat immune-mediated diseases, there is little information regarding the effects of exogenous GCs on the gut microbiota. Several studies in rodent models have demonstrated GC-induced changes in the intestinal (fecal) microbiota that vary among species, subjects, and study design [3,10,11,12,13,14,15,16,17,18]. A prevailing pattern that emerges is that exogenous GCs increase the abundance of Firmicutes [14,19,20] and Actinobacteria [14,19,21], while the abundance of Bacteroidetes decreases [12,14,19,20]. Importantly, these data were generated using different GCs (prednisone, prednisolone, dexamethasone, and hydrocortisone), suggesting a common mechanism of action. In one clinical trial involving dogs diagnosed with chronic inflammatory enteropathy (CIE, formally known as inflammatory bowel disease), therapy with prednisone and metronidazole was associated with the altered intestinal abundance of select bacterial groups [22]. In another study, oral administration of prednisone (1 mg/kg) for 14 days to healthy dogs had no effect on fecal bacterial diversity or composition [8]. While these previous canine studies investigated the association between GC administration and the fecal microbiota, the impact of GCs on the gastric and duodenal mucosal microbiota of healthy dogs is unknown.

The objective of this study was to investigate the effects of immunosuppressive doses of prednisone (2 mg/kg/d PO) compared to the placebo on the fecal and mucosal microbiota of healthy dogs. We hypothesized that high-dose prednisone administration would alter the gastric and duodenal mucosal microbiota, but not the fecal microbiota, of healthy dogs.

## 2. Materials and Methods

### 2.1. Animals

Archived gastric and duodenal mucosal biopsies obtained from 12 healthy laboratory-reared dogs that participated in a randomized, double-blinded, placebo-controlled trial were analyzed [23]. The animal use/clinical trial protocol was reviewed and approved by the IACUC committee at the University of Tennessee, Knoxville (protocol number 2283).

### 2.2. Study Design

Dogs were stratified by age and then randomized to either the placebo or prednisone treatment group. Dogs were acclimated to their surroundings for 14 days (days −13 to 0), followed by a 28-day treatment period (days 1–28). All dogs received water ad libitum and were fed a balanced canine commercial dry ration (20% of metabolizable energy as protein) throughout the treatment schedule. Placebo group dogs were administered lactose-containing gelatin capsules (LetCo Medical, Decatur, AL, USA), and glucocorticoid group dogs received prednisone (West-Ward Pharmaceuticals Corp., Eatontown, NJ, USA) at a dosage of 2 mg/kg q24 h PO. All treatments were administered in small meatballs (Purina ONE SmartBlend Healthy Puppy Lamb and Long Grain Rice; Nestle, Switzerland) once daily by an individual blinded to treatment groups. Naturally voided feces were collected on treatment days −2 to 0, 12 to 14, and 26 to 28.

Esophagogastroduodenoscopy was performed, and multiple endoscopic biopsies from the stomach and duodenum were obtained at each of the three timepoints, as previously described [24]. Mucosal biopsies were placed in 10% neutral-buffered formalin, routinely processed, and paraffin-embedded as a tissue block for H&E histopathologic and fluorescence in situ hybridization (FISH) analyses.

### 2.3. Mucosal Microbiome Analysis

Formalin-fixed embedded tissue sections were prepared for fluorescence in situ hybridization (FISH), as previously described [24]. Briefly, sections were mounted on glass slides, deparaffinized, and then air-dried prior to hybridization. Cy-3- or FITC-labeled FISH probes were reconstituted with DNAse-free water and diluted to a working concentration of 5 ng/μL. Specific probes targeting the most common bacterial isolates from the stomach and small intestine [25], as well as the universal bacterial probe Eub338, were applied to tissues (Supplemental Table S1). The probes selected for the stomach targeted *Helicobacter* spp., *Streptococcus* spp., and *Lactobacillus* spp., while the probes for the small intestine targeted *Clostridium* spp., *Enterobacteriaceae*, and *Bacteroides* spp. Tissue sections were bathed in 30 μL of DNA–probe mix for 12 h at 54 °C, washed and rinsed, allowed to air dry, and then mounted with SlowFade Gold mounting media (Life Technologies, Carlsbad, CA, USA). The quantification of fluorophore-labeled (Cy3 or FITC) bacterial populations present within the adherent mucus was performed using Metamorph^®^ automated software, (MetaMorph v7.8) as previously described [24,26].

### 2.4. Fecal Microbiome Analysis

Genomic DNA was extracted from 100 mg of feces for each timepoint using a commercially available DNA extraction kit (PowerSoil R^®^, Mo Bio, Carlsbad, CA, USA), according to the manufacturer’s instructions. Amplification and sequencing of the V4 variable region (primers 515F/806R) of the 16S rRNA gene were performed on a MiSeq (Illumina) at MR DNA, as described previously. The software QIIME was used for the processing and analysis of sequences. The raw sequence data was de-multiplexed, and low-quality reads were filtered using the default parameters. Chimeric sequences were detected using USEARCH and removed prior to further analysis, and the sequences were then assigned to operational taxonomic units (OTUs), using an open-reference OTU-picking protocol in QIIME against the Greengenes database. The OTU table was rarefied to 35,000 sequences per sample.

Beta diversity for level 2 investigation included *Actinobacteria* spp., *Bacteroidetes* spp., *Deferribacteres* spp., *Firmicutes* spp., *Fusobacteria* spp., *Proteobacteria* spp., and *Tenericutes* spp. Additional investigation for amplicon sequence variants was performed with a focus on *Clostridium* clusters IV and XIV (*Lactobacillus* spp., *Paraprevotella* spp., *Bacteroides* spp., *Helicobacter* spp., *Actinomyces* spp., *Bifidobacterium* spp., the family *Lachnospiraceae, Ruminococcus* spp., *Megamonas* spp., *Blautia* spp., *Roseburia* spp., *Coprococcus* spp., *Clostridium* spp., the family *Ruminococcaceae, and Ruminococcus* spp.). The oligonucleotide primers and probes, as well as respective annealing temperatures of the primers, are summarized in Supplemental Tables S1 and S2.

Quantitative PCR was performed for selected bacterial groups (total bacterial, *Faecalibacterium* spp., *Turicibacter* spp., *Streptococcus* spp., *Escherichia coli*, *Blautia* spp., *Fusobacterium* spp., and *Clostridium hiranonis*) using extracted DNA, as has been previously described [27] (Supplemental Table S2). Briefly, 2 µL of normalized DNA (final concentration: 5 ng/µL) was combined with 5 µL of a DNA-binding dye (SsoFast EvaGreen supermix; Bio-Rad Laboratories, Hercules, CA, USA), 0.4 µL each of a forward and reverse primer (final concentration: 400 nM), and 2.6 µL of PCR water to achieve a total reaction volume of 10 µL. Data were expressed as the log amount of DNA (fg) for each bacterial group per 10 ng of isolated total DNA.

The dysbiosis index (DI) was calculated from the quantitative PCR analyses. The DI summarizes the fecal abundance of 7 bacterial taxa and total bacteria. A DI > 2 indicates a significant shift in the overall microbiota diversity, <0 is normal and indicates no significant shifts in the overall microbiota diversity, and 0–2 indicates mild-to-moderate shifts in the overall microbiota diversity, as shown previously [27]. The higher the dysbiosis index, the greater the severity of dysbiosis.

### 2.5. Statistical and Data Analysis

Tabular data were organized by probe used and treatment group. Descriptive statistics were generated for each response measure. Normality of the data was assessed visually by histograms and Q–Q plots. Global changes in microbiota communities (beta diversity) between individuals were determined using unweighted Unifrac distance metrics; principal coordinates analysis (PCoA) plots and rarefaction curves were plotted using QIIME software. The ANOSIM function in PRIMER 6 (PRIMER-E Ltd., Ivybridge, UK) was used to compare beta diversity metrics across time and between treatment groups [28].

Mixed model, split-plot, repeated measures ANOVAs that include the fixed effects of treatment, time, and treatment-by-time interaction were used to compare quantitative bacterial counts for each bacterial genus in the feces, Shannon indices, goods coverage, the Chao 1 metric, and the dysbiosis index between treatment groups. Dogs nested within groups were included as a random effect in all mixed model analyses. Model assumptions regarding normally distributed residuals were verified with the Shapiro–Wilk test for normality and QQ plots. Model assumptions regarding equality of variances were verified with Levene’s Test for Equality of Variances. Differences in least squares means were determined for bacteria counts and relative abundances with a significant main effect or interaction terms. Only bacteria taxa that were present in at least 50% of dogs in ≥1 group at ≥1 timepoint were included in the statistical analyses. Non-normally distributed data were logarithmically or rank-transformed, as necessary, to meet underlying statistical assumptions. If logarithmic transformation was required, 0.05 was added to all values. *p*-values were corrected for multiple comparisons on each phylogenetic level for microbiome evaluations using Benjamini & Hochberg’s False Discovery Rate (FDR). Comparison of the numbers of mucosal bacteria was performed with GraphPad Prism 9 (version 9.4.1) (https://graphpad.com/; accessed on 2 September 2022) using one-way ANOVA, followed by Šídák’s multiple comparisons test. A *p*-value of <0.05 was considered significant for all analyses. Publicly accessible software packages (http://www.qiime.org; MedCalc 15.8: MedCalc, Ostend, Belgium; SAS 9.4 release TS1M3: SAS Institute Inc., Cary, NC, USA) were used for all microbial community analyses.

## 3. Results

### 3.1. Animals

The placebo group included two castrated males, one intact male, and three intact females with a median age of 3.5 years (range 2–6 years), whereas the prednisone group was comprised of two castrated males, two intact males, and two intact females with a median age of 3 years (range 2–6 years). Pooled samples for the microbiome analysis for two dogs at timepoint 2 (one from each group) were comprised of only two fecal samples, since they could not be collected separately. The clinical, clinicopathologic, and gastrointestinal changes associated with the administration of high-dose prednisone to the healthy dogs of this study have been previously reported [29].

### 3.2. Mucosal Microbiota

**Figure 1:**
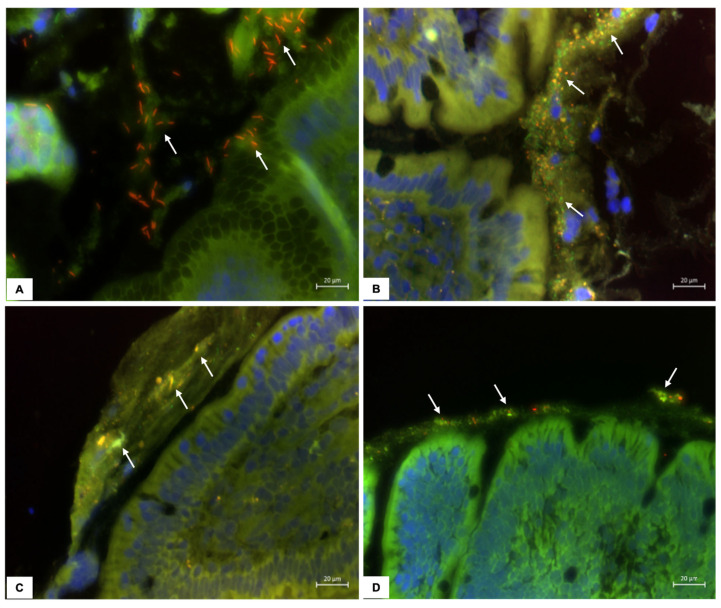
Three-color FISH identifies mucosal bacteria present in canine gastric and duodenal endoscopic biopsies following treatment (day 28) with either the placebo or prednisone. Specific bacterial groups (*Helicobacter*, *Clostridia*, *Bacteroides*, and *Enterobacteriaceae*) hybridizing with Cy3 appear orange (arrows). All other bacteria hybridizing with the universal probe (EUB-FITC) appear green. DAPI-stained mucosal epithelia stain blue. (**A**) Gastric *Helicobacter* from a prednisone group dog, (**B**) duodenal *Bacteroides* from a prednisone group dog, (**C**) duodenal *Enterobacteriaceae* from a placebo group dog, and (**D**) duodenal *Clostridia* from a placebo group dog. All images are at 400× magnification.

**Figure 2:**
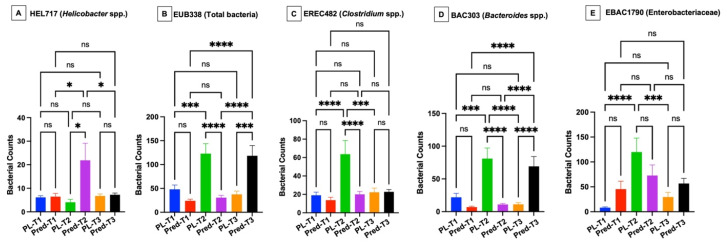
Numbers of mucosal bacteria in gastric and duodenal biopsies of healthy dogs over the treatment schedule. Data are expressed as mean ± standard deviation. Gastric *helicobacters* (**A**), duodenal total bacteria (**B**), *Clostridia* (**C**), *Bacteroides* (**D**), and *Enterobacteriaceae* Panel (**E**). PL = placebo group, Pred = prednisone group, T1 = timepoint 1, T2 = timepoint 2, and T3 = timepoint 3. **** significantly different at *p*-value < 0.0001, *** significantly different at *p*-value < 0.001, and * significantly different at *p*-value < 0.05. ns = no significant difference.

The microbiota of the gastric mucosa was colonized by *Helicobacter* spp., located within the gastric mucus layer, representing over 95% of the total bacterial load in the biopsy specimens (Figure 1). While there was no difference in the numbers of *Helicobacter* spp. between dog groups at timepoint 1, prednisone group dogs had significantly increased (*p* < 0.05) numbers of helicobacters at timepoint 2 compared to placebo group dogs and compared to prednisone group dogs at timepoints 1 and 3 (Figure 2A). There was no difference in the numbers of helicobacters between dog groups at timepoint 3. Very few (<10 bacteria/10 representative fields) *Streptococci* and *Lactobacilli* were visualized in the gastric tissues.

The mucosal microbiota in the duodenum of healthy dogs was patchy in distribution and most abundant in adherent mucus (Figure 1). There were no differences in the numbers of bacteria between dog groups at timepoint 1. However, the numbers of total bacteria, *Clostridia*, *Bacteroides*, and *Enterobacteriaceae* were significantly increased (*p* < 0.05) in placebo group dogs compared to prednisone group dogs at timepoint 2 and compared to the bacterial numbers in placebo group dogs at timepoint 1 (Figure 2B–E). The numbers of *Bacteroides* in placebo group dogs at timepoint 2 exceeded (*p* < 0.05) their numbers at timepoint 3. The total numbers of bacteria and *Bacteroides* were greatest (*p* < 0.05) in prednisone group dogs at timepoint 3 as compared to placebo group dogs at timepoint 3 and compared to prednisone group dogs at timepoints 1 and 2 (Figure 2B,D).

### 3.3. Fecal Microbiota

Alpha diversity did not differ between groups or among timepoints for the Shannon index, observed amplicon sequence variant (ASV), or Chao 1 metrics (*p* ≥ 0.05 for all, respectively), as noted in Supplemental Table S3. Beta diversity and additional investigation for amplicon sequence variants did not differ significantly between groups or among timepoints (R = −0.105, *p* = 0.991 for weighted UniFrac distance) (Supplemental Table S4).

**Figure 3:**
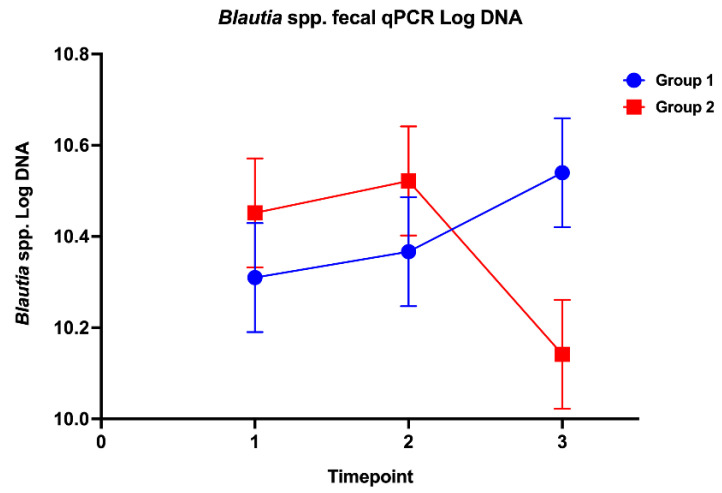
*Blautia* spp. fecal qPCR log DNA change between animals receiving the placebo (group 1) versus prednisone (group 2) at baseline (timepoint 1, days −2–0), midway through the study period (timepoint 2, days 12–14), and at the end of the 28-day treatment schedule (timepoint 3, days 26–28).

Abundances for the taxa evaluated by qPCR remained within their respective reference intervals for all dogs at all timepoints. However, a significant group by time interaction (*p* = 0.042, F-value 3.72) was noted for *Blautia* spp. Based on post hoc analysis, this reflected a significantly decreased *Blautia* spp. abundance at timepoint 3 for dogs in the prednisone group (Figure 3). The dysbiosis index was within the reference interval for all samples, with no significant differences between groups or among timepoints (Supplemental Figure S1).

## 4. Discussion

Glucocorticoids are powerful anti-inflammatory, immune-modulating drugs for the treatment of inflammatory conditions (chronic enteropathies, rhinitis, and immune-mediated hemolytic anemia), as well as orthopedic, dermatologic, and ophthalmologic disorders [8,30,31,32,33,34,35]. While the common side effects of polyuria, polydipsia, bodyweight gain or loss, and GI mucosal injury are often clinically apparent, GCs have also been shown to alter the composition of the intestinal (fecal) microbiota [11,12,13,14,15]. Our data suggest that dogs administered immunosuppressive doses of prednisone showed alterations in select groups of the gastric and duodenal mucosal microbiota. Compared to placebo group dogs, dogs administered prednisone had increased numbers of helicobacters in the stomach and increased numbers of total bacteria and Bacteroides in the duodenum over the treatment period. Alpha and beta diversity of the fecal microbiota, as well as the DI, did not differ between treatment groups or timepoints. However, the fecal abundance of *Blautia* spp. was decreased at timepoint 3. In a separate study, the effects of metronidazole or prednisolone (1 mg/kg PO q 24 h) on the fecal microbiome of healthy dogs were investigated before (day 0) after (day 14) treatment and 14 and 28 days after drug cessation [8]. No effect of prednisone on the fecal microbiota was observed. Like previous reports [36], metronidazole significantly altered the composition of some bacterial groups on day 14 compared with other timepoints. The data obtained in this earlier clinical study and our current research suggest that different GCs used short term at either anti-inflammatory or immunosuppressive levels do not significantly alter the fecal microbiota of healthy dogs.

The gut microbiota (e.g., bacteria, archaea, fungi, protozoa, and viruses) plays an important role in host health and disease [37,38,39]. It forms an essential component of the intestinal epithelial barrier, contributes to the host metabolism, protects against pathogens, and influences development of the mucosal immune system [8,36]. Previous studies have identified a core intestinal microbiota composed of several phyla, including *Actinobacteria*, *Bacteroidetes*, *Firmicutes*, *Fusobacteria*, and *Proteobacteria*, in the fecal samples of healthy dogs [36,40]. Within this core community, several major taxa are considered beneficial and belong to the phylum *Firmicutes*, such as *Clostridia* and *Bacilli*, many of which are important short-chain fatty acid producers, including *Faecalibacterium* [37]. Other members of the resident microbiota, such as the family *Enterobacteriaceae*, are normally present in the small intestine in small numbers but are increased in the feces and mucosa of dogs with chronic inflammatory enteropathy (CIE) due to intestinal inflammation and associated dysbiosis [37,41].

The microbiota in the stomach of healthy dogs is predominated by several different *Helicobacter* spp. [42]. Different studies indicate a high prevalence rate (e.g., 50–100%) of non-*H. pylori* gastric helicobacter-like organisms (GHLO) in healthy dogs and cats, questioning the potential pathogenic role of these organisms in gastritis [43]. Furthermore, clinical signs of gastric disease are infrequently present in most infected dogs, and studies show an inconsistent association between the presence of GHLOs and histopathologic parameters [44]. The literature on the mucosal microbiota of healthy dogs is less extensive. Most studies have reported the mucosal microbiota of healthy (control) dogs compared to the microbiota in dogs with chronic gastrointestinal diseases. In separate studies, the mucosal microbiota of dogs with CIE was investigated in endoscopic biopsies [45] or cytologic brushings [25] of the duodenum by performing 454-pyrosequencing or evaluating gene clone libraries. The general patterns of mucosal dysbiosis in diseased dogs include reduced biodiversity with increased numbers of *Proteobacteria* and decreased numbers of *Fusobacteria*, *Clostridia*, and *Bacteroidaceae*. Using FISH, invasive *Escherichia coli* (*E. coli*) are found within inflamed colonic mucosa of dogs with granulomatous colitis [46,47,48]. Other FISH studies have shown that ileal and/or colonic tissues of dogs with CIE harbor a dysbiosis characterized by increased numbers of mucosal Enterobacteriaceae and *E. coli* compared to control tissues [24,26,46,49]. Moreover, depletion of the colonic surface and crypt bacteria (e.g., *Helicobacter* spp. and *Akkermansia* spp.) were observed in dogs with CIE [49].

Two FISH studies have evaluated the treatment effects of GCs on the mucosal microbiota in dogs with chronic gastroenteritis. Atherly et al. [50] used a six-probe array (targeting *Bifidobacterium* spp., *Enterobacteriaceae*, *Faecalibacterium* spp., *Lactobacillus* spp., *Streptococcus* spp., and total bacteria) to investigate the mucosal microbiota of dogs with CIE treated with an elimination diet and immunosuppressive doses of prednisone for 8 weeks. The spatial distribution of the mucosal bacteria was significantly different following prednisone therapy, with increased numbers of *Bifidobacteria, Faecalibacterium,* and *Streptococci* present within the adherent mucus. A second study, using a similar trial design and probe set, compared the treatment of dogs with CIE using prednisone or a multi-strain probiotic [26]. The results showed that prednisone-treated dogs had increased numbers of mucosal *Bifidobacteria* compared to dogs receiving probiotics. The mechanisms responsible for modulation of the intestinal microbiota by GCs remain poorly defined but may include changes in the mucus (qualitative and quantitative), an altered production of antimicrobial peptides and secretory IgA, increased intestinal permeability, and modulation of the NOD-like receptor family pyrin domain containing 6 (NLRP6) inflammasome [10,51,52,53].

The hypothalamic–pituitary–adrenal (HPA) axis and endogenous GC secretion are functionally influenced by a normal microbiota, as evidenced by the exaggerated response of acute stress in different rodent models [54,55,56,57,58]. For example, germ-free rats demonstrate altered neuroendocrine and behavioral responses to acute stress as compared to specific pathogen-free male rats, accompanied by increased levels of corticosterone in plasma ^55^. Germ-free mice undergoing restraint have increased levels of corticosterone, indicative of stress associated with the restraint procedure [59]. Other animal models have shown that neonatal rats exposed to probiotics early after birth are protected against elevated HPA responses and intestinal barrier dysfunction [60]. Stress has been shown to increase serum corticosteroid levels, alter the murine microbiome, and decrease the levels of intestinal *lactobacilli* while increasing the levels of *E. coli* and *pseudomonas* [61,62]. Stress also increases the expression of bacterial virulence genes, which can negatively affect intestinal function [61]. Finally, the role of environmental stress in a large animal model has been recently investigated. The prebiotic gallnut tannic acid was shown to ameliorate the stress-induced inflammatory response, fecal dysbiosis, and altered metabolome in laboratory beagles by targeting the intestinal microbiota [63]. These collective findings in different animal models suggest a complex interaction between endogenous and exogenous GCs and the GI microbiota, whose details remain poorly defined.

While not the aim of the present study to report, endoscopic lesions of gastric bleeding, erosions, and ulceration were observed in healthy dogs receiving immunosuppressive dosages of prednisone compared to the placebo group. Other studies have reported similar findings of gastric mucosal hemorrhages, erosions, and ulcers in healthy dogs administered prednisone (2 mg/kg q24 h PO) alone or in combination with aspirin (2 mg/kg q24 h PO) for 28 days, with the greatest gastric mucosal injury scores seen in dogs receiving combination treatment [23]. In a separate study where healthy dogs were administered a placebo, prednisone (2.3 mg/kg q24 h PO), or prednisone and aspirin (0.5 mg/kg q24 h PO) for 27 days, there were no significant differences in gastroduodenal lesion scores between the different treatment groups [64]. Finally, the concurrent administration of meloxicam (0.1 mg/kg, SC, q12 h for 3 days) and dexamethasone (0.25 mg/kg, SC, q12 h for 3 days) to healthy dogs was more likely to cause gastric mucosal lesions than meloxicam administration alone [65].

There are some limitations in our study, with the first being the small sample size of the groups, with only six dogs in each cohort. The short study duration of 28 days may have underestimated the treatment effects of high-dose GCs on the intestinal microbiota if they were administered for a longer period. Our selection of FISH probes used to identify mucosal bacteria may have missed alterations in other microbial community members affected by diet and GC administration. While the same diet (having an identical macronutrient composition) was fed to both cohorts, it remains possible that other dietary factors may have affected the mucosal bacterial composition in the prednisone group dogs [66].

In conclusion, immunosuppressive doses of prednisone administered to healthy dogs have little effect on the fecal microbiota, including the DI. In contrast, the dogs administered prednisone showed variable but significant alterations in mucosal gastric *Helicobacters*, duodenal total bacteria, and *Bacteroides* over the treatment period. The microbiota from mucosal samples more clearly reflect the underlying microbial alterations in response to high-dose prednisone treatment, as compared to the fecal samples.

## Data Availability

The data presented in this study are available in the tables and figures.

## Supplementary Materials

The following supporting information can be downloaded at: https://www.mdpi.com/article/10.3390/vetsci12030216/s1. Supplemental Table S1: Probes, target bacteria, and sequences used for fluorescence in situ hybridization. Supplemental Table S2: Target bacteria, oligonucleotide primers, and annealing temperatures used for quantitative PCR analysis. Supplemental Table S3: Dysbiosis index and alpha diversity results for dogs in the treatment trial. Median and (range) results for feces collected at baseline (timepoint 1), midway through study period (timepoint 2), and at the end of the treatment schedule (time point 3) in placebo and prednisone group dogs. Supplemental Table S4: Median values of examined taxa with ranges for feces collected at baseline (timepoint 1), midway through study period (timepoint 2), and at the end of the treatment schedule (time point 3) in placebo and prednisone group dogs. Supplemental Figure S1. Summary data for all bacterial taxa in the DI.
