# Supplementary material for: Effects of High-Dose Prednisone on the Gastrointestinal Microbiota of Healthy Dogs
Source: Vet Sci. 2025 Mar 2;12(3):216. doi: 10.3390/vetsci12030216 (PMC11946374; doi:10.3390/vetsci12030216)

Supplemental Table S1:

Probes, target bacteria, and sequences used for fluorescence in situ hybridization.

**Gastric Mucosa**		
**Probe**	**Target**	**Sequence (5' →3')**
EUB338	Eubacteria	GCT GCC TCC CGT AGG AGT
HEL717	*Helicobacter* spp.	AGG TCG CCT TCG CAA TGA GTA
LAB158	*Lactobacillus* spp.	GGT ATT AGC ATC TGT TTC CA
STR493	*Streptococcus* spp.	GTT AGC CGT CCC TTT CTG G
**Duodenal Mucosa**		
**Probe**	**Target**	**Sequence (5' →3')**
EUB338	Eubacteria	GCT GCC TCC CGT AGG AGT
EREC482	*Clostridium* spp.	GCT TCT TAG TCA RGT ACC G
EBAC1790	*Enterobacteriaceae*	CGT GTT TGC ACA GTG CTG
BAC303	*Bacteroides* spp.	CCA ATG TGG GGG ACC TT

Supplemental Table S2:

Target bacteria, oligonucleotide primers, and annealing temperatures used for quantitative PCR analysis.

**Target**	**Primer Type**	**Sequence (5'-3')**	**Annealing (°C)**
Eubacteria	Forward<br>Reverse	CCTACGGGAGGCAGCAGT<br>ATTACCGCGGCTGCTGG	59
Blautia spp.	Forward<br>Reverse	TCTGATGTGAAAGGCTGGGGCTTA<br>GGCTTAGCCACCCGACACCTA	56
Clostridium hiranonis	Forward<br>Reverse	AGTAAGCTCCTGATACTGTCT<br>AGGGAAAGAGGAGATTAGTCC	50
Escherichia coli	Forward<br>Reverse	GTTAATACCTTTGCTCATTGA<br>ACCAGGGTATCTAATCCTGTT	55
Faecalibacterium spp.	Forward<br>Reverse	GAAGGCGGCCTACTGGGCAC<br>GTGCAGGCGAGTTGCAGCCT	60
Fusobacteria spp.	Forward<br>Reverse	KGGGCTCAACMCMGTATTGCGT<br>TCGCGTTAGCTTGGGCGCTG	51
Streptococcus spp.	Forward<br>Reverse	TTATTTGAAAGGGGCAATTGCT<br>GTGAACTTTCCACTCTCACAC	54
Turicibacter spp.	Forward<br>Reverse	CAGACGGGGACAACGATTGGA<br>TACGCATCGTCGCCTTGGTA	63

Supplemental Table S3:

	Timepoint 1 (-2-0)		Timepoint 2 (Day 12-14)		Timepoint 3 (Day 26-28)	
	Placebo	Prednisone	Placebo	Prednisone	Placebo	Prednisone
Dysbiosis index	-1.2419 (-5.6989 to -0.4461)	-1.8018 (-5.4178 to -0.7216)	-1.6452 (-5.4863 to -0.2481)	-1.1085 (-5.3181 to -0.3925)	-1.5504 (-4.5900 to 0.6519)	-1.5409 (-6.4500 to 0.3593)
Shannon index	6.7909 (5.5850 to 8.1077)	6.3324 (5.5785 to 7.5929)	6.5316 (5.5338 to 8.1317)	6.3422 (5.4707 to 7.2463)	6.1431 (5.0148 to 8.2624)	6.4783 (5.4846 to 8.2969)
Observed ASV	391 (194 to 792)	353.5 (205 to 657)	408 (237 to 862)	349 (189 to 643)	299 (149 to 655)	353 (169 to 667)
Chao1 metric	393.5147 (196.625 to 809.0123)	359.3538 (207.4 to 660.4872)	416.0057 (256 to 904.0779)	355.3403 (191.1538 to 663.5091)	315.2571 (151.3333 to 656.1579)	357.6917 (169.5 to 668.8571)

Dysbiosis index and alpha diversity results for dogs in the treatment trial. Median and (range) results for feces collected at baseline (timepoint 1), midway through study period (timepoint 2), and at the end of the treatment schedule (time point 3) in placebo and prednisone group dogs.

Supplemental Table S4:

**qPCR**	Placebo (Group 1)			Prednisone (Group 2)			fdr P-value		
	Timepoint 1 (-2-0)	Timepoint 2 (12-14)	Timepoint 3 (26-28)	Timepoint 1 (-2-0)	Timepoint 2 (12-14)	Timepoint 3 (26-28)	Group	Time	Group*Time
Dysbiosis Index	-1.2419 (-5.6989 to -0.4461)	-1.6452 (-5.4863 to -0.2481)	-1.5504 (-4.59 to 0.6519)	-1.8018 (-5.4178 to -0.7216)	-1.1085 (-5.3181 to -0.3925)	-1.5409 (-6.45 to 0.3593)	0.23	0.29	1.13
Uniserval	11.025 (10.63 to 11.08)	11.095 (10.72 to 11.33)	11.215 (10.87 to 11.52)	11.045 (10.7 to 11.25)	11.255 (10.79 to 11.52)	11.095 (10.09 to 11.23)	0.03	1.34	3.39
Faecalibacterium	5.965 (4.45 to 7.24)	6.23 (4.89 to 7.13)	5.95 (4.68 to 7.22)	6.08 (4.84 to 7.17)	6.28 (4.57 to 6.92)	6.42 (4.53 to 6.86)	0.01	0.17	0.29
Turicibacter	7.745 (7.0 to 8.02)	7.76 (7.08 to 8.1)	7.815 (7.22 to 8.17)	7.755 (7.67 to 8.06)	8.075 (7.57 to 8.42)	7.755 (7.66 to 8.02)	1.83	2	2.45

Streptococcus	6.855 (4.22 to 7.72)	6.51 (4.48 to 8.03)	7.365 (4.5 to 8.58)	6.65 (4.48 to 8.12)	7.25 (4.5 to 7.82)	7.445 (3.88 to 8.58)	0.07	1.07	1.03
E. Coli	4.055 (1.8 to 6.33)	4.405 (1.91 to 6.34)	4.11 (0.88 to 5.45)	3.42 (1.04 to 5.44)	4.395 (0.88 to 7.01)	4.075 (2.6 to 5.87)	0.07	0.55	1.45
Blautia	10.25 (9.96 to 10.76)	10.35 (10.11 to 10.73)	10.53 (10.34 to 10.78)	10.465 (10.23 to 10.66)	10.53 (10.18 to 10.76)	10.24 (9.18 to 10.73)	0.11	0.41	3.72
Fusobacterium	8.485 (6.61 to 9.12)	8.605 (7.72 to 9.45)	7.945 (7.23 to 9.2)	8.51 (6.9 to 9.36)	8.975 (6.59 - 9.74)	8.795 (6.34 to 9.39)	0.01	1.11	0.28
Clostridium hiranonis	6.51 (6.38 to 6.86)	6.115 (6.04 to 6.83)	6.61 (6.31 to 7.15)	6.68 (6.21 to 7.0)	6.435 (6.0 to 7.07)	6.34 (6.16 to 7.14)	0.22	2.64	1.98
**Level 2**									
Actinobacteria	1839 (912 to 13014)	1214 (770 to 2546)	1515 (843 to 2176)	1630.5 (1187 to 2095)	1481.5 (1268 to 2640)	1794.5 (812 to 3023)	0.9972	0.8828	0.9972
Bacteroidetes	870 (16 to 6864)	926.5 (69 to 4005)	139.5 (28 to 7260)	222.5 (20 to 3511)	358 (2 to 1794)	1133 (54 to 5527)	0.9972	0.9972	0.5176
Deferribacteres	0 (0 to 28)	0.5 (0 to 20)	0 (0 to 72)	0.5 (0 to 24)	1 (0 to 5)	2.5 (0 to 43)	0.9972	0.9972	0.9972

Firmicutes	29271 (21077 to 31135)	29091 (24745 to 31078)	30482.5 (18418 to 31701)	30344 (20433 to 31345)	29383 (28071 to 31290)	27540.5 (22221 to 31728)	0.9972	0.9972	0.8828
Fusobacteria	486.5 (0 to 3327)	730 (211 to 1722)	128.5 (0 to 3929)	250.5 (18 to 5902)	722 (4 to 1648)	1420 (0 to 2122)	0.9972	0.9972	0.8828
Proteobacteria	176.5 (57 to 1912)	226 (62 to 997)	49 (9 to 2002)	51 (3 to 964)	153 (41 to 420)	194 (101 to 883)	0.9972	0.9972	0.2981
Tenericutes	1 (0 to 47)	0 (0 to 41)	0 (0 to 88)	0 (0 to 21)	0 (0 to 0)	0.5 (0 to 30)	0.9972	0.5176	0.9972
**ASV**									
Lactobacillus	1447.5 (0 to 4639)	2653 (219 to 11805)	2777 (96 to 4307)	730.5 (0 to 16362)	6728 (267 to 15539)	2207 (448 to 10199)	0.9849	0.9849	0.9849
Bacteroidetes_Paraprevotella	0 (0 to 0)	3.5 (0 to 35)	0 (0 to 50)	0 (0 to 29)	0 (0 to 25)	0 (0 to 48)	0.9849	0.6653	0.6653
Bacteroidetes_Bacteroides	469 (16 to 3512)	298.5 (66 to 1543)	93 (1 to 3335)	132.5 (12 to 2224)	206.5 (2 to 537)	381 (52 to 1867)	0.9849	0.9849	0.6653
Proteobacteria_Helicobacter	0 (0 to 57)	54 (0 to 91)	2 (0 to 53)	0 (0 to 3)	15.5 (0 to 46)	0 (0 to 56)	0.9849	0.6653	0.9849

Actinobacteria_Actinomyces	12.5 (0 to 29)	3.5 (0 to 15)	3.5 (0 to 43)	11 (3 to 21)	1.5 (0 to 14)	6 (0 to 23)	0.9849	0.6653	0.9849
Bifidobacterium	156 (0 to 767)	306 (0 to 461)	251 (34 to 796)	713.5 (68 to 913)	559.5 (60 to 1775)	277.5 (29 to 1921)	0.8404	0.744	0.744
Firmicutes_Lachnospiraceae_Ruminococcus	341.5 (117 to 640)	366.5 (170 to 613)	260.5 (167 to 374)	407.5 (167 to 803)	380.5 (140 to 463)	297 (216 to 512)	0.9849	0.6653	0.9849
Firmicutes_Veillonellaceae_Megamonas	130 (0 to 505)	232 (0 to 1161)	6.5 (0 to 362)	25.5 (0 to 341)	98 (0 to 227)	115.5 (0 to 498)	0.9849	0.744	0.6653
Firmicutes_Lachnospiraceae_Blautia	705 (250 to 1444)	545.5 (288 to 1702)	684.5 (439 to 1459)	688 (381 to 1820)	624 (367 to 1433)	629 (347 to 1656)	0.9849	0.9849	0.9849
Firmicutes_Lachnospiraceae_Roseburia	0 (0 to 10)	0 (0 to 46)	0 (0 to 16)	1 (0 to 88)	0 (0 to 0)	0 (0 to 43)	0.9849	0.9849	0.6653
Firmicutes_Lachnospiraceae_Coprococcus spp.	27 (0 to 116)	34.5 (0 to 142)	30 (0 to 146)	0 (0 to 122)	4.5 (0 to 54)	0 (0 to 153)	0.9849	0.9849	0.9849
Firmicutes_Lachnospiraceae_Clostridium perfringens	47 (0 to 235)	27 (0 to 523)	0 (0 to 184)	41 (0 to 305)	5.5 (0 to 91)	31 (0 to 767)	0.9849	0.9849	0.744
Firmicutes_Ruminococcaceae_Ruminococcus	0 (0 to 10)	0 (0 to 46)	0 (0 to 16)	1 (0 to 88)	0 (0 to 0)	0 (0 to 43)	0.9849	0.9849	0.6653

Median values of examined taxa with ranges for feces collected at baseline (timepoint 1), midway through study period (timepoint 2), and at the end of the treatment schedule (time point 3) in placebo and prednisone group dogs.

Supplemental Figure S1. Summary data for all bacterial taxa in the DI.

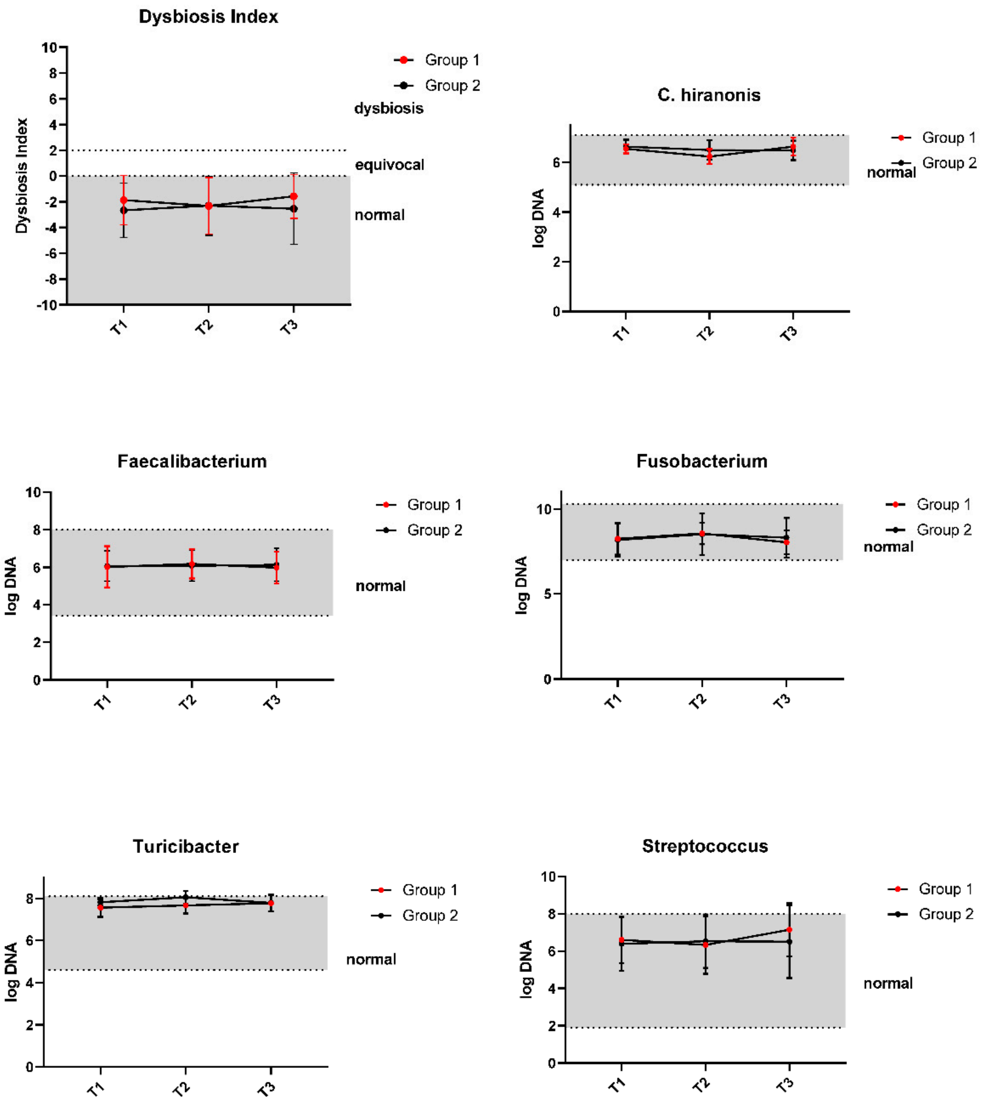

Supplement: Supplementary file 1 [file vetsci-12-00216-s001.zip › vetsci-3447127-supplementary.pdf]
